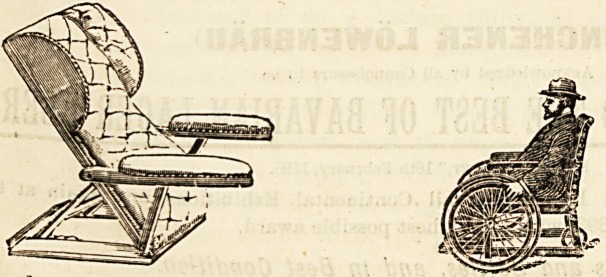# Practical Departments

**Published:** 1900-10-06

**Authors:** 


### OTHER HOSPITALS.

### PRACTICAL DEPARTMENTS.

### NOVELTIES IN INVALID AND HOSPITAL FURNITURE, FITTINGS, &c.

(By Our Commissioner.)

#### Messrs. Doulton & Co. (Lim.).

Messrs. Doulton's sanitary "specialities for hospitals" are, perhaps, almost too well known to need comment, but a very attractive new catalogue of fittings, just issued, seems to call for a word of notice. The advance in every kind of sanitary fitting has been rapid of late, and fresh and important improvements are constantly being brought out. Messrs. Doulton are always in the van of progress in their particular departments, and their glazed ware and enamelled work have attained wide and well-deserved notoriety. Cast-iron baths, of which Messrs. Doulton show so many designs, specially adapted for hospital use, can now be coated with vitreous enamel, both inside and out, giving a surface as smooth as porcelain, though less chilling and less expensive, while more durable, and no kind of bath is better for hospital and institution purposes. Here are enumerated also all the well-known varieties of slop sinks and drainers, with sprays for flushing bed-pans; one of these sinks is fitted with a brass-bound glass cover, made tight by means of a rubber joint, which entirely precludes the danger in infectious illness of any harmful matter splashing beyond the sink, while by an ingenious arrangement every time the sink is used the cistern discharges automatically, completely cleansing every part. The glazed ware lavatories and sinks for operating rooms, worked by treadle arrangements, which fulfil every requirement of modern asepticism are well known, as also are the improved "Simplicitas" closets, with non-absorbent seats, silent arrangements, and other advantages. These and many another hospital speciality will be found in the showrooms on the Albert Embankment, where they may be seen in action.

#### Messrs. Farmer, Lane & Co.

With the return of so many sick and wounded men from South Africa there has naturally been a considerable demand upon all kinds and descriptions of invalid chairs and couches and other paraphernalia suitable to the needs of our crippled heroes, and Messrs. Farmer, Lane & Co., New Oxford Street, W., have plenty of all varieties on show. The chair, a self-propelling and carrying variety of the ordinary invalid chair, of which an illustration here appears, has proved very useful for its double purpose, being so arranged as practically to take the place of two chairs. It is fitted with rubber-tyred bicycle wheels for easy locomotion, and with shifting steel front handles and folding back handles for convenience in carrying patients up or down stairs. The top part of the chair lifts off for carrying, and trestles are supplied to support it while the under, or wheel portion, is waited for.

A very useful, simple, and inexpensive carrying litter, made of birch, polished or stained, with cane seat and back, and legs made to unscrew, should be mentioned, while a folding carrying chair, also made of birch and cane, with folding handles, &c., deserves commendation. Messrs. Farmer, Lane & Co.'s well-known reclining and self-propelling merlin chairs, for private or hospital use, are always in demand, and in their show rooms in New Oxford Street every design, from the simple and inexpensive to the most elaborate, are to be seen. One of our illustrations shows one of the ever-useful back-rests of which this firm makes a speciality, equally a comfort in home sick-room or hospital ward. Other appliances which should be noted are the "walking machines" or go-carts, intended for the use of patients who have wholly or partially lost the use of their limbs. These are mounted on rubber castors or bicycle-wheels, with crutches adjustable to any height to suit the needs of tall or short persons, and are ingenious aids in such cases. Hospital authorities should note that Messrs. Farmer, Lane & Co. offer special prices to hospitals and institutions.

#### The Quaker Bath Cabinet (Gem Supplies Company).

For those people who find Turkish or vapour baths helpful and beneficial, but are deterred from making use of such a luxury because of the expense and trouble, the cabinet bath brought out by the Gem Supplies Company, Bishop's Court, Chancery Lane, E.C., should prove a very acceptable substitute. Its framework is of galvanised steel in four sections, connected by hinges, and rubber-lined and coated, giving plenty of interior space when adjusted for use, and folding up into a small compass when closed. A chair and a specially-designed stove and cover, heating with methylated spirit, complete the accessories, and provide all that is needful for a chamber vapour bath, while its construction is simple enough to make it possible for anyone to manage the whole affair unaided. The price is 30s.

#### Sponges for the Million.

Messrs. Cresswell Brothers & Schmitz, sponge importers, &c., Red Lion Square, W.C., are an enterprising firm, and have established a large business in that very essential article of daily life—sponges—having their own divers and boats on the sponge fisheries of the Mediterranean, collecting sponges also from the islands by their own steamers, thus supplying the English market direct from the source. We should recommend application to Messrs. Cresswell for their catalogue, which, besides giving an interesting account of the "Natural and Commercial History of the Sponge," details the many varieties of honeycomb, Turkey, Florida, and other sponges which are to be obtained from them at moderate prices and of the best quality.

#### The Taunton Patent "Di-ag-nl" Spring Mattress.

It is satisfactory to be able to draw attention to a new and reliable spring mattress, brought out as a speciality for hospital and institution use by Messrs. John & Joseph Taunton, Sherbourne Road, Birmingham. There are several points specially deserving of notice in this mattress, with its "anti-sagging mesh." First, perhaps, the perfectly level surface, so important an essential in any bed intended for the use of sick people. This result is achieved by placing the springs diagonally, or in a direct line with the links, so that a direct strain is secured, and the tautness and resiliency of the centre increased very considerably. The tension is evenly distributed, every spring being in play, not merely those in the middle where the weight is placed. The surface is elastic, with the yielding qualities of a woven mesh, yet minus its disadvantages. Messrs. Taunton are willing to guarantee these mattresses for 20 years' wear, and, further, they are ready to send sample bedsteads for the inspection of institutional authorities when any quantity are required.

A Cheap Bicycle.

To anyone who is wanting a bicycle at a low price the offer now being made by Symonds' London Stores, New North Street, W.C., will certainly prove tempting. For the small sum of £4 19s. 9d. this firm offers a "first-class 1901 model cycle," "complete in all respects," full particulars of which may be found in the descriptive catalogues to be had upon application. It is an introductory offer only, and it is stated that the machines will be raised to twelve guineas in the spring.

(Poulton and Noel, Belgravian Steam Works, Brewery Road, London, N.)

We have received from the above firm samples of certain of their invalid specialities—namely, home-made beef-tea, essence of chicken, and essence of beef. The home-made beef-tea, which is described as of double strength—a somewhat ambiguous epithet—appeals to us as a very excellent example of the manufacturers' interpretation of home-made beef-tea. Home-made beef-tea as generally met with, hardly constitutes a standard to be emulated; its chief virtue lies in having been freshly prepared. The beef-tea prepared by Poulton and Noel possesses every merit except the last-named. From an artistic and gastronomic point of view it is infinitely superior to domestic cooks' interpretation of the same commodity. We would suggest, however, that by preserving the same in bottles instead of tins public prejudice would be more easily humoured. The latter suggestion applies equally to the meat essences, which are otherwise of first-rate quality and eminently suited to the capricious appetite of invalids and convalescents.

## Figures and Tables

**Figure f1:**